# Effectiveness of Biochar Obtained from Corncob for Immobilization of Lead in Contaminated Soil

**DOI:** 10.5696/2156-9614-9.23.190907

**Published:** 2019-07-23

**Authors:** Alfonso Rodriguez, Daniela Lemos, Yessika T. Trujillo, Julián G. Amaya, Laura D. Ramos

**Affiliations:** 1 Universidad de la Sabana, Cundinamarca, Colombia; 2 Pure Earth, New York, NY; 3 R3 Environmental Technology Colombia, SAS, Bogota, Colombia

**Keywords:** biochar, soil remediation, gasification, contamination, lead, pH, moisture, retention, adsorption, carbon sequestration, heavy metals

## Abstract

**Background.:**

Recent studies have explored the potential for using biochar as a soil amendment in agriculture. However, it can also be used as a gentle remediation option for contaminant reduction. Biochar is a by-product obtained from the pyrolysis of biomass (organic matter). It is known for its long-lasting chemical properties, wide surface area values, and carbon-richness, which make it an efficient method for the immobilization of organic and inorganic contaminants such as heavy metals.

**Objective.:**

The aim of the present study was to analyze the efficiency of biochar, obtained from the gasification of corncob, for the immobilization of lead in contaminated soils.

**Methods.:**

In the present study, biochar from corncob was used as an amendment for soil contaminated with lead (extracted from the municipality of Malambo, Colombia) in order to estimate its ability to immobilize leaching lead. A comparison laboratory test applied a modified biochar produced with a 10% hydrogen peroxide chemical treatment. In addition, a pot experiment was done with both biochar by sowing seeds of Pennisetum clandestinum for 33 days. During this period, plant growth was measured for the different amendments of biochar concentrations.

**Results.:**

Laboratory tests indicated that unmodified biochar obtained a maximum retention of 61.46% of lead, while the modified biochar obtained only 44.53% retention. In the pot experiments, the modified biochar indicated high germination and growth of seeds (up to 89.8%).

**Conclusions.:**

Although the lead immobilization in soil was positive for both cases, the use of soil with high concentrations of lead (167.62 g/kg) does not indicate biochar's effectiveness for purposes of comparison with the current United States Environmental Protection Agency (USEPA) limit value (400 ppm for bare soil in urban play areas). Therefore, further studies are recommended using soil with lower lead concentration levels.

**Competing Interests.:**

The authors declare no competing financial interests. One author is an employee of Pure Earth.

## Introduction

Contamination of soils by both organic and inorganic pollutants is an issue worldwide, and environmentally friendly alternatives for addressing this problem are being investigated.^1^ A number of new alternative techniques utilize an in-situ, low-invasive approach involving plants (with or without chemical additives) to reduce contaminant transfer to the environment by direct extraction of pollutants (clean up) or by soil stabilization (using biological or chemical processes). Collectively, these techniques are sometimes referred to as “gentle” remediation options.^2^

Biochar is a solid material that is rich in carbon and is synthesized by hydrothermal carbonization or by slow pyrolysis of biomass.^3^ These processes produce biochar with a remarkable alkaline nature that is favorable for the treatment of acidic soils. The processes principally involve the thermal decomposition of biomass such as oil palm, cottonseed husk, orange peel, bamboo, and various organic wastes under anaerobic conditions.^4^–^8^ The application of biochar as a means of remediation and soil strengthening has been studied over the last decade due to its efficiency and cost effectiveness. These days, over one thousand studies around biochar and soil enhancement are published each year, and scientific interest is growing.^9^–^12^ Biochar's physicochemical properties, such as porosity, surface area, pH, conductivity, and structure are determining factors in its impact and interaction with soil that help to increase crop yields and carbon sequestration, reduce soil greenhouse gases, and favor the immobilization of organic and inorganic (including metallic) pollutants.^3^,^4^,^11^,^13^–^21^

This research aims to analyze the efficiency of biochar obtained from the gasification of corncob (after the corn kernels have been removed) with the immobilization of lead in contaminated soil.

## Methods

The tests were performed on soil contaminated with high levels of lead from the municipality of Malambo, located on the north coast of Colombia. Additionally, a chemical modification of biochar was performed, and the results obtained from both types of biochar were compared. Physicochemical tests were carried out in order to evaluate the changes generated in the soil using the two types of biochar. In addition, a Pennisetum clandestinum pot experiment was conducted parallel to the previously mentioned tests to analyze the effect of biochar on plant growth.

Hydrogen peroxide was used to produce the modified biochar sample, which increased the functional groups containing oxygen and aided metal sorption.^19^,^22^ In comparison with other techniques, the hydrogen peroxide modification is cost effective, easily accessible, the decomposition products H_2_O and O_2_ are environmentally friendly, and at a 10% concentration it has a greater absorption compared to commercial alternatives. Chemical activation methodologies with potassium hydroxide, carbon dioxide and steam current physical techniques require high temperatures, approximately 800°C, which increases the process costs and risks.^18^,^23^

### Sampling

Thirty (30) kg of soil was collected superficially from an abandoned lead smelter in La Bonga Village in the Malambo municipality. The location is a public health concern due to the associated lead poisoning cases in the surrounding community. The biochar residue was obtained from the gasification of corncob, which was used to produce renewable energy.^8^

**Abbreviations**
*USEPA*: United States Environmental Protection Agency

### Soil characterization

Soil texture and structure were determined using a physical analysis procedure provided by the Ministry of Agriculture and Forestry of Alberta, Canada.^24^ A sample of 100 g of homogenized soil was required for the analysis. Subsequently, a chemical analysis provided an adequate characterization of the soil, as well as values of electrical conductivity, pH, volatile solids, and humidity. These properties were measured according to Banos *et al*.^25^ Moisture was measured using the gravimetric method.^26^ The concentration of lead in the soil indicates the amount of lead available for each planting modification. Lead concentration was determined through an analysis of total metals by inductively coupled plasma mass spectrometry, which employs a microwave-assisted acid digestion method according to standards proposed by the United States Environmental Protection Agency (USEPA).^26^,^27^

### Biochar characterization

The conditions to conduct the pyrolysis process include raising the temperature range to 130–600°C and forming part of the gasification process of the corncob. The pyrolysis process consisted of four principal stages: first, drying the biomass inside the hopper of the gasifier; second, using tar removal to eliminate the mass percentage that was not considered in the design; third, provide devolatilization or decomposition of the biomass in its constituent elements, and fourth, biomass gasification.^8^ Before gasification, the sample was brought to a humidity of less than 30% and a particle size between 1 and 4 cm. The equipment used was a fixed-bed, downdraft gasifier (ALL Power Labs, California, USA). The pyrolysis process is detailed in a thesis on renewable energy from the University of La Sabana by Martinez.^8^ The white rachis of corn used in the present study is mainly composed of cellulose (40–50%), hemicellulose (20–30%), and lignin (10–40%).^8^,^28^,^29^ After gasification, the biochar was sieved in order to avoid large granules or chunks. The final result was a homogeneous fine powder.

### Biochar chemical and physical analysis

The physical-chemical biocarbon analysis used 10 g of biochar and 200 ml of water. Previously suggested methods were used to determine the electrical conductivity and pH of the biochar.^25^ The surface area was calculated by the Brunauer-Emmett-Teller method, which deducts the surface area by desorption and adsorption of N2 at 77 K.^30^ Lastly, the final analysis was carried out following ASTM D5373-14, method A.^31^,^32^

### Biochar modification

Biochar modification was performed by dissolving 261 g of biochar in 1740 ml of hydrogen peroxide at 10%, giving rise to an exothermic reaction. The mixture was then left to stand for 2 hours at a temperature of 22°C. Finally, it was washed with distilled water and dried in an oven at 80°C.^19^

### Execution of the sowing test (pot experiment)

Pennisetum clandestinum, a grassforming specie that can spread progressively, was chosen for sowing in the pot experiment. Pennisetum clandestinum adapts easily to humid tropics or subtropics, especially at higher elevations and in high fertility soils.^33^

**Figure 1:**
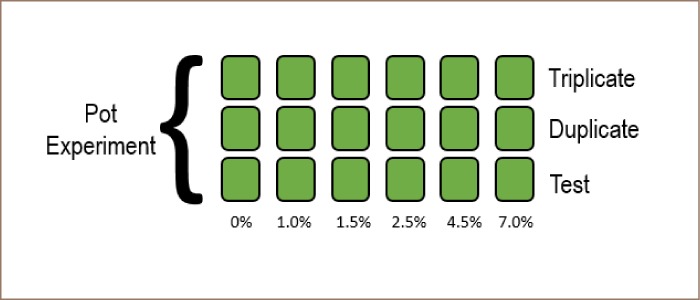
Distribution of pot experiment

Two pots were chosen for sowing experiments and growth monitoring, one pot for modified biochar and other for non-modified biochar, both with equal measures. Each were filled with 80 g of contaminated soil and a specific percentage of biochar, homogenized by sieving. The biochar-soil mixtures consisted of 0%, 1%, 1.5%, 2.5%, 4.5% and 7% biochar concentrations and in triplicates, as shown in Figure 1.

Application began by depositing 3/8^th^ of the mixture's total volume. Then, the sowing was carried out by the furrow method, which involved placing the Pennisetum clandestinum seeds in a linear pattern, followed by covering the seeds with the remaining mixture.^34^ Spatial uniformity factors and depth were taken into account for sowing.^35^–^37^ Twenty (20) seeds of Pennisetum clandestinum were deposited and evenly spaced. The application was then repeated, starting with depositing 3/8^th^ of the mixture's total volume and followed by 20 more seeds spread on the surface before being covered with the remaining mixture. Therefore, each pot had a total of 40 seeds. Each pot was watered every 24 hours and provided 50 ml of water per cell. The pots were placed under a controlled laboratory environment. Follow-up for the two sowing tests occurred over 33 days. The sowing parameters were the same for both biochar types.

### Detection of lead reduction

The concentration of lead reduction after biochar amendment was determined through an analysis of total metals by inductively coupled plasma mass spectrometry, which employs a microwave assisted acid digestion method according to standards proposed by the USEPA.^27^

## Results

Table 1 presents the baseline soil characterization results, unmodified biochar treatment results, and the modified biochar treatment results (modified with 10% hydrogen peroxide).

**Table 1:** Biochar/Soil Characterization Results

	**Soil**	**Biochar**	**Modified biochar**
**Conductivity (mS/cm)**	0.732	10.15	6.86
**pH**	5.53	9.42	10.22
**Surface area - BET (m2/g)**	-	80.14	196.4
**Texture**	Sandy clay loam	Silty clay	Silty clay
**Structure**	Single grain	Granular structure	Granular structure
**Volatile solids mg/kg**	2.8657	-	-
**Humidity**	5.3%	-	-

Abbreviations: BET, Brunauer Emmet and Teller.

The non-modified biochar contained carbon, hydrogen, nitrogen and oxygen in percentages of 76.3%, 2.18%, 10.53%, and 1.31%, respectively. The non-modified biochar was obtained from corncob organic matter under a pyrolysis temperature between 130°C and 600°C.

### Lead availability in soil as a function of biochar concentration

**Figure 2:**
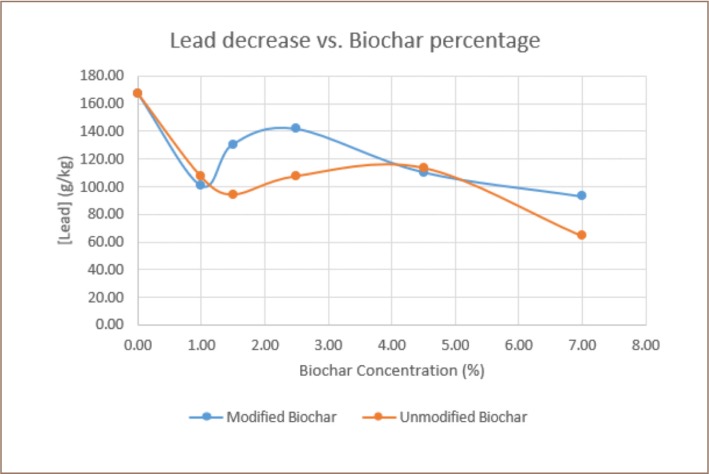
Lead availability in soil as a function of biochar concentration

The first pot experiment tested unmodified biochar and the second experiment tested modified biochar. The results of both are presented in Figure 2.

Statistical analysis indicated differences between biochar types. The unmodified biochar treatment presented a lower soil lead concentration. However, the concentration of lead from the modified biochar was still acceptable as the variation coefficient for each percentage was lower than 25%, as shown in Table 2.

**Table 2:** Lead Retention in Soil by Biochar Type

**Biochar %**	**Lead in soil (g/kg) with modified biochar**	**Lead in soil (g/kg) with unmodified biochar**	**Average**	**Standard deviation**	**CV**
**0.00**	167.62	167.62	167.62	0.00	0.00
**1.00**	100.81	107.45	104.13	4.70	0.05
**1.50**	130.62	94.48	112.55	25.55	0.23
**2.50**	141.75	107.80	124.78	24.01	0.19
**4.50**	110.44	113.77	112.10	2.35	0.02
**7.00**	92.98	64.60	78.79	20.07	0.25

Abbreviation: CV, coefficient of variation.

### Sowing results

**Figure 3:**
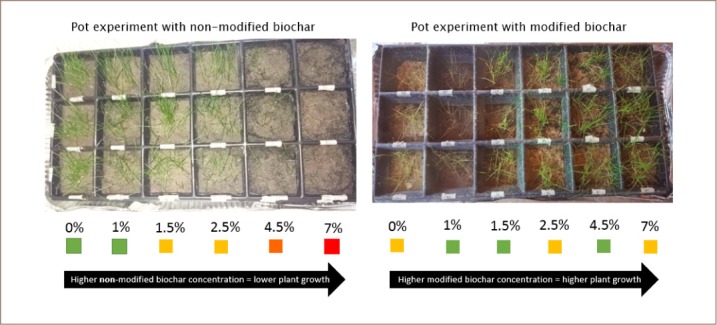
Photo report of growth after 33 days for modified and non-modified biochar

The 33-day growth monitoring showed behavior as an exponential function of time for each biochar concentration. Regarding the unmodified biochar pot experiment, it was observed that as the biochar concentration increased, there was a significant negative growth effect (*Figure 3*). Conversely, increases in modified biochar concentrations showed a positive effect on growth with the 7% concentration providing the best results. This result is in contrast to the unmodified biochar results where the 7% concentration of unmodified biochar provided the lowest growth.

Seed germination occurred after 12 days in the unmodified biochar concentrations and after 10 days in the modified biochar concentrations.

**Figure 4:**
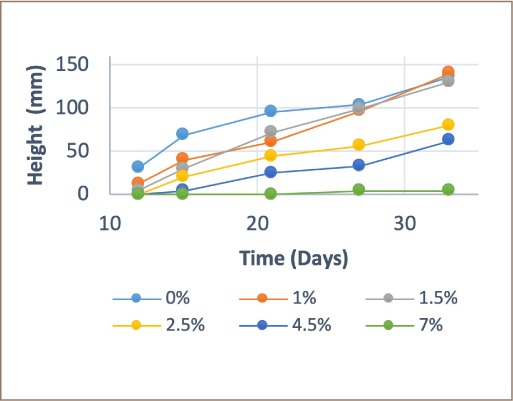
Growth of Pennisetum clandestinum using unmodified biochar

**Figure 5:**
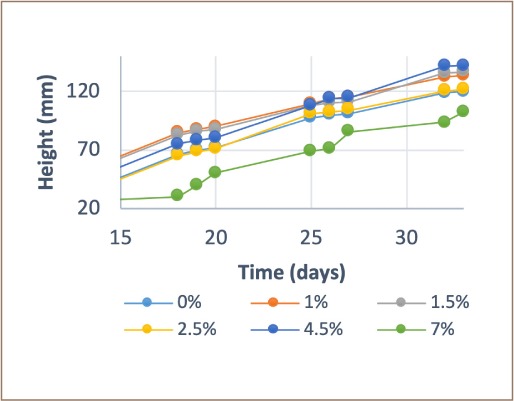
Growth of Pennisetum clandestinum using modified biochar

Figure 4 and 5 show the growth trend that occurred during sowing. Unmodified biochar provided greater growth in the concentrations containing 0%, 1% and 1.5%, while 7% presented the poorest growth. Modified biochar provided significant growth at all concentrations with the greatest growth at 4.5% and 7%. Development significantly improved in cells with 1.5%, 2.5%, 4.5%, and 7% concentrations of modified biochar, and growth was improved by 4.6%, 34.2%, 52.1%, and 89.8%, respectively. The cell containing 1% of modified biochar showed low growth with just a 4.7% difference in height.

Regarding lead contamination, sowing with unmodified biochar caused a reduction in lead concentration of up to 61.46%. The reduction in lead contamination using modified biochar was 44.53%. Although the reduction percentages are significant, it was not possible to bring lead contamination levels below the USEPA permissible standards of “400 parts per million (ppm) of lead in bare soil in children's play areas or 1200 ppm average for bare soil in the rest of the yard.”^38^,^39^ These standards were determined in 2001 according to the maximum level of lead to which a child could be safely exposed, given that children represent the most vulnerable population in terms of health.^39^,^40^

## Discussion

The unmodified biocarbon had a higher metal retention of 61.46% compared to the modified biocarbon retention of 44.53%. However, the desired outcomes of the pot experiments were inversely related: the modified biochar provided higher plant growth while the unmodified biochar provided lower plant growth. Yet both types of biocarbon generated positive results in the soil's physical and chemical properties and in sowing growth.^41^ Generally, the type of biocarbon dictates the resulting inhibition of microbial activity that causes the immobilization of nitrogen that is vital for plant growth.^42^,^43^ It is important to note that the same raw material can produce different types of biocarbon, each with a different nitrogen content that impacts the biocarbon-soil interaction.^11^

The biochar-treated soil was composed of single grain, structureless soil. Characteristics of structureless soil include increased runoff and high risk of water erosion. Use of this soil could be the cause for the limited growth seen in the present study. Biochar has a granular structure and can provide a better growing environment for plant roots, likely due to its high carbon content that contributes to the plant life cycle.^24^ Carbon provides greater water retention and permeability in soil, so the addition of biochar can substantially improve water retention capacity.^4^,^44^,^45^

Modified biochar increased pH and decreased the solubility of metals (with the exception of metalloids). Hence, biochar with basic pH levels is known for yielding higher crop productivity in acidic soils.^11^,^46^ Soil alkalization is vital to understanding the growth and immobilization of pollutants, because the pH of biochar directly affects pH levels in soil.^3^,^45^ For a highly alkaline biochar and acidic soil sample, a limestone amendment effect normally occurs, as the acidity of the soil will decrease, leading to a significant increase in crop growth.^46^ However, not all types of biochar generate the same growth effect because the results are related to the type of species being cultivated.^11^

The best growing conditions were observed in cases of moderate or low biochar addition, similar to the results of previous studies.^47^–^49^ The conditions (pH, conductivity, immobilization capacity, surface area) of growth are improved by modification, as observed in this study's modified biochar treatment results *(Figure 4 and 5).*^46^,^50^–^53^

Different remediation patterns were observed with unmodified biochar where pH increased, but growth decreased considerably. This may have been due to biochar's high adsorption capacity, capturing essential nutrients from the plant, such as phosphorus and sulfur. Therefore, the availability of these nutrients in the soil may decrease and adversely affect plant development.^54^,^55^–^57^ Furthermore, growth was inhibited by the unmodified biochar amended-soil's high levels of conductivity, where conductivity deviated between acceptable growth values of 0–0.8 ms/cm.

Table 3 indicates that the biochar modification process contributed to decreased and regulated soil conductivity compared to the unmodified biochar, where conductivity values were too high. Consequently, the latter substrate was not a propitious environment for growth.^58^,^59^ Organic matter content is an important variable in the development and absorption of nutrients, where the appropriate level for sowing grasses is between 8% and 12%, and the minimum soil organic matter content for basic plant development is 2%.^60^–^63^ However, this is not ideal for sowing pastures. The soil organic matter content for the current study's soil sample was 3.9%, and although this is not the ideal value for grass growth, it meets the minimum percentage of organic matter for growth to occur.

**Table 3:** Biochar Pot Experiments

**Biochar (%)**	**pH**	^**^pH	**Electrical conductivity (mS/cm)**	^**^Electrical conductivity (mS/cm)	**Lead (g/kg)**	^**^Lead (g/kg)	**Lead retention (%)**	^**^Lead retention (%)	**Maximum growth (mm)**	^**^Maximum growth (mm)
0.00	5.53	5.53	0.73	0.73	167.62	167.62	_	-	150	126
1.00	7.36	7.89	2.27	1.15	107.45	100.81	35.90	39.86	140	142
1.50	8.01	8.01	3.38	1.54	94.48	130.62	43.63	22.08	130	140
2.50	8.87	8.98	4.43	1.96	107.80	141.75	35.69	15.43	80	147
4.50	9.40	9.93	5.15	2.20	113.77	110.44	32.13	34.11	70	160
7.00	9.72	10.14	9.50	3.23	64.60	92.98	61.46	44.53	31	160

^**^ The results obtained with modified biochar

Other studies of biochar chemical modification show the potential for increased biochar surface area, which is an important indicator of its adsorption capacity.^64^ A study by Tang *et al*. showed that a wheat straw biochar sample had a surface area of 4.5m^2^/g. A graphene modification increased the sample's surface area to 17.3m^2^/g and, in turn, improved mercury retention by 31.6%.^65^ Thus, the surface area of the graphene-modified biochar was approximately 3.84 times greater than that of the unmodified biochar. This outcome was reflected in a hydrogen peroxide modification, where the surface area was approximately 2.45 times greater than that of the unmodified biochar.^4^

The relationship between oxygen and carbon indicates how aromatic or hydrophilic the surface of biochar can be.^3^ The ratio obtained in the present study was 0.138. Thus, the surface of the biochar is more aromatic than hydrophilic, which is due to a greater carbon extension and loss of functional groups that present a polar nature at high temperatures.^66^ Evidence of this was provided by the elemental analysis that indicated the high aromatic carbon content. Both the organic matter of origin and the pyrolysis conditions are significant in the final carbon concentration.^19^ The biochar used in this study had a carbon concentration of 76.3%, which is higher than other types of biochar. For example, the biochar of spruce wood has a 51.21% carbon concentration and biochar from corn waste at a temperature of 350°C contains 67.5% carbon, however, at 600°C it has a higher content (79.0%).^3^,^67^

In a study by Ahmad *et al.* a comparison was done between several biochar feedstock from broiler waste, buffalo weed, canola waste, cottonseed coatings, orange peel shells, peanut shells, poultry manure, sewage sludge, and wood waste, among others.^4^ Elemental analysis values were in the range of 20.19% - 95.30% for carbon, 0.42%–7.25 for hydrogen, 0.01%–46.80% for oxygen and 0.04%–10.21% for nitrogen. Thus, the obtained biochar of corncob values are within the expected ranges.^3^

The results of soil lead concentration were favorable with regard to lead retention capacity when using unmodified biochar *(Table 3).* The retention of lead from modified biochar did not present any significant changes to justify chemical modification costs, as the samples were not below the permissible limit (400 ppm).^39^,^68^ This was due to the extremely high contamination levels that were found in the study's soil.

Soil conductivity dropped by 50.66%, from 2.27 mS/cm to 1.15 mS/cm (in cells with soil and biochar at 1%). Both biochars created healthy soil conditions (0–0.8 mS/cm).

## Conclusions

Although the use of biochar as a soil amendment is still considered an option for strengthening the organic matter and increasing the growth of species, there is not enough scientific evidence to support its use as a remediation method in contaminated soils or as an alternative intervention.

Further studies are needed using soils with more environmentally viable concentrations of lead and biochar comprised of different types and combinations of biomass.
